# North Dakota

**Published:** 1896-04

**Authors:** T. D. Hinebauch

**Affiliations:** Secretary; Fargo, N. D.


### NORTH DAKOTA VETERINARY MEDICAL ASSOCIATION.

THE fourth annual meeting convened December 4, 1895, at Hotel Dakota, Grand Forks. The following members responded to roll-call: Drs. Taylor, Turcot, Crewe, La Pointe, Shepperd, Hinebauch. Visitors present were Drs. Miller, O'Connor, and Graftegraph.

The morning session was taken up principally with the report of the Treasurer and Secretary and the reading of a paper by Dr. Hinebauch on "The Use of Electricity in the Practice of Veterinary Medicine and Surgery." The paper was discussed at considerable length, a number of the veterinarians present having previously assisted the writer in diagnosing several cases of difficult lameness by means of the battery.

The afternoon session was opened by the President's address, and followed by another paper on the subject of "Millet Disease in Horses." This paper was a continuation of the one presented two years previously. In the discussion which followed Dr. Turcot remarked that he had been somewhat skeptical regarding the influence of millet as a disease-producing food. He said that the evidence of the experiment, however, convinced him that millet had more or less of an influence in the producing of pathological conditions.

The society is in a very healthy condition, having now a total membership of twenty. There are but twenty-one graduated veterinarians in the State, hence we think we can boast of a larger percentage of membership than possibly any other State society.

The society congratulates itself upon being able to have a law passed at the last session of the Legislature which hereafter will practically control the practice of veterinary medicine and surgery in the State. Although the law was not all the society wished, yet it was all that we could expect to have passed. The Legislature was thoroughly canvassed as soon as it had been elected, and we could not do better than to have the present law passed.

SECTION 1615. *Qualifications of Veterinarians.* Each person practising veterinary medicine, surgery, or dentistry in any of its departments in this State shall possess the qualifications required by this article; provided that any person who has practised veterinary medicine, surgery, or dentistry as a profession in this State for three years immediately preceding the passage and approval of this article, and who shall be a citizen of the United States or shall have declared his intention to become such, shall be deemed eligible to registration, and shall receive a certificate upon presentation of a sworn affidavit and letters of recommendation from five reputable freeholders in his locality, or upon presentation of a diploma from a legally authorized veterinary school, college, or university, if made before July 1, 1895.

SEC. 1616. *Board of Examiners; How Appointed; Term.* The Governor shall appoint a Board of Examiners within thirty days after the passage of this article, to be known as the State Board of Veterinary Medical Examiners. Such Board shall consist of three practising veterinarians, who shall each be the holder of a diploma granted by a legally authorized veterinary school, college, or university, who shall hold office one for one year, one for two years, and one for three years after such appointment, or until their successors are appointed. Thereafter each year the Governor shall appoint one member of said Board to fill the vacancy occasioned by the expiration of the term of office of those previously appointed, and is further authorized to fill such vacancies as may occur.

SEC. 1617. *Organization of Board.* Said Board shall elect a President, Secretary, and Treasurer. It shall have a common seal, and the President and Secretary shall have power to administer oaths. Said Board shall hold meetings for the examination of candidates on the second Wednesday of April and October of each year, and such other meetings as may be deemed necessary, at such time and place as the Board may appoint; no session to exceed two days. The Board shall issue a certificate of qualification to all applicants who shall pass the required examination, and who shall be citizens of the United States, or shall have legally declared their intention to become such, and to all applicants who are eligible to registration under Section 1615, signed by the President and Secretary of the Board. Such certificate or diploma shall be conclusive as to the right of the lawful holder of the same to practice veterinary medicine, surgery, or dentistry in this State. Said Board shall keep a record of all the proceedings thereof, and also a record or register of each applicant for a license, together with his age, name, and time spent in the study and practice of veterinary medicine, surgery, or dentistry; and, if a graduate, the name and location of the school, college, or university granting such diploma. Said books and records shall be *prima facie* evidence of all the matter therein recorded.

SEC. 1618. *Permit to Practice.* Any person wishing to practise veterinary medicine, surgery, or dentistry, who is qualified under Section 1621, may apply to the President of the Board of Examiners for a permit to practise. The President shall upon payment of five dollars, if satisfied that the applicant is qualified aud a suitable person, issue to him a permit to practise until the next meeting of the Board, and such permit shall have the same force as a certificate from the Board, but shall expire upon the adjournment of the next meeting of the Board of Examiners.

SEC. 1619. *Diplomas and Certificates.* Persons presenting diplomas or certificates for registration shall pay to the Treasurer of said Board a fee of ten dollars in advance; and the fees received by said Board shall be paid over to the State Treasurer within thirty days after receipt of same. Said fees shall constitute a special fund for the payment of the expenses of said Board of Examiners. Each member of said Board shall receive from the State Treasury all necessary travelling expenses actually incurred in attending such meetings.

The Secretary of the Board shall certify to the State Auditor after each meeting of the Board the amount due each member for necessary expenses in attending such meetings, and other necessary expenses of the Board. The State Auditor shall thereupon issue his warrant on the State Treasurer for such sum, provided there has been a sufficient amount paid into the treasury in fees to redeem said warrants; but if there is not an amount equal to said certified expenses to the credit of such fund, he shall issue his warrant for the amount in the said special fund, and deficiencies in the payment of said expenses may be made up from subsequent receipts.

SEC. 1620. *Misdemeanor to Practise, etc.; When.* Any person who either: 1. Practises veterinary medicine, surgery, or dentistry in this State without compliance with the provisions of this article; or, 2. Wilfully and falsely claims or pretends to have or hold a certificate of registration issued by such Board; or, 3. Wilfully and falsely, with intent to deceive the public, claims or pretends to be a graduate of or to hold a diploma granted by a legally authorized veterinary school, college, or university, is guilty of a misdemeanor, and upon conviction is punishable by a fine of not less than fifty nor more than one hundred dollars, and in case of non-payment of such fine the person so offending shall be liable to imprisonment for a period not exceeding six months. All fines received under this article shall be paid into the common-school fund of the county in which such conviction takes place.

SEC. 1621. *Examination.* All persons commencing the practice of veterinary medicine, surgery, or dentistry in this State after the passage and approval of this act shall be graduates of a legally authorized veterinary school, college, or university, and shall subject themselves to such examination as the Board may require.

SEC. 1622. *Certificates Recorded.* Every person holding a certificate from the Board of Examiners shall have it recorded in the office of the Register of Deeds in the county in which he resides within thirty days after the date of said certificate, and the record shall be indorsed thereon. Any person removing to another county to practise shall record within thirty days the certificate in a like manner in the county to which he removes, and the holder of the certificate shall pay to the Register of Deeds a fee of one dollar for making the record.

SEC. 1623. *Gratuitous Services.* Gratuitous service in cases of emergency, in the dehorning of cattle or castration of animals, shall not be construed as coming within the meaning of this article.

SEC. 1624. *Witnesses; Expert Fees.* Any person complying with the provisions of this article shall be entitled to expert fees as a witness in all civil actions relating to the veterinary profession.

T. D. HINEBAUCH,
FARGO, N. D. Secretary.

